# Cell-Free Nucleic Acids: Physico-Chemical Properties, Analytical Considerations, and Clinical Applications

**DOI:** 10.3390/diagnostics13132312

**Published:** 2023-07-07

**Authors:** Abel J. Bronkhorst, Stefan Holdenrieder

**Affiliations:** Munich Biomarker Research Center, Institute of Laboratory Medicine, German Heart Center, Technical University Munich, 80636 Munich, Germany

Human body fluids are rich sources of cell-free nuclear material, which exhibits unique characteristics. Cell-free DNA (cfDNA) molecules originate from diverse sources, including nuclei, mitochondria, different cell types, tumors, different organs (including transplanted and damaged organs), fetuses, the environment, invading pathogens, and the microbiome. Despite their short half-lives, they are continuously replenished. They possess numerous genetic and epigenetic features that reflect the identity of their source and specific molecular, physiological, and pathological processes. They demonstrate biological activities involved in normal biological functions (e.g., immunomodulation) and pathology (e.g., oncogenesis and metastasis). Furthermore, the collection of cfDNA samples from body fluids, such as blood or urine, is relatively non-invasive, enabling serial sampling. Therefore, the cfDNA population in body fluids provides real-time information on host and meta-genomic changes, which can be explored for various purposes.

Unsurprisingly, systematic profiling of cfDNA is increasingly recognized for its potentially profound impact on modern genetics and molecular medicine. For example, cfDNA molecules can serve as highly specific surrogate molecular markers for diagnosing and monitoring a wide range of pathologies and diseases. CfDNA can also be used to investigate changes in physiological states, such as during exercise. Furthermore, the longitudinal characterization of cfDNA over extended periods will allow for the mapping of temporal genomic changes in individuals or populations in response to factors like environmental exposures, physiological states, and pathologies. Leveraging this information not only has the potential to revolutionize personalized medicine in the future but also provides insights into poorly understood mechanisms underlying pathological events and other significant cellular and biological processes.

The articles featured in this Special Issue cover a broad spectrum of research topics within the field, shedding light on the diverse properties and potential applications of cfDNA. However, they also emphasize the challenges that must be addressed to fully unlock the potential of cfDNA characterization.

## 1. A Brief History of cfDNA Research

CcfDNA was first discovered in 1948, preceding the establishment of molecular biology as a discipline. While initially regarded as trivial and even dismissed as pseudoscience, the biological and clinical significance of cfDNA became apparent two decades later when abnormal cfDNA features were found to correlate with various diseases. This realization catalyzed a surge of interest in cfDNA, leading to the emergence of a dedicated research field. Today, cfDNA stands as one of the most interesting biological molecules, driving intensive investigation and shaping the development of advanced technologies, analytical methods, and bioinformatics procedures.

In this rapidly expanding field, it is crucial to not only focus on the cutting edge but to also grasp the broader contours of research. In their review paper, Gahan et al. underscore the importance of this broader perspective by reflecting on the historical progression of cfDNA research [1]. Their concise account emphasizes two key aspects: first, the intertwined nature of cfDNA research with the history of molecular biology, gene structure, and function; and second, the existence of overlooked concepts in the literature, such as the potential biological and pathological functions of cfDNA, its role as a messenger molecule, and its potential contribution to animal evolution. Revisiting these ideas through the lens of contemporary scientific understanding and technical capabilities promises to be an intriguing endeavor.

## 2. Preanalytical Considerations for cfDNA Analysis

Despite recent advancements, optimizing, standardizing, and harmonizing preanalytical workflows for cfDNA remains a challenging and evolving task. Several papers in this Special Issue address various preanalytical issues and provide some insights into these challenges.

Diaz et al. evaluated the performance of Streck Cell-Free DNA blood collection tubes (cfDNA BCTs) in comparison to standard K2EDTA tubes for blood sample collection and storage in cancer patients. They assessed the circulating tumor DNA (ctDNA) yield, genomic DNA contamination, and mutation status of specific genes. The study revealed similar levels of ctDNA yield and gDNA contamination between both tube types, regardless of storage duration (up to 3 days for cfDNA BCTs and 6 h for K2EDTA tubes). Additionally, the mutational loads of samples stored in both tube types remained consistent across different cancer patient cohorts and concentrations. These findings support the use of cfDNA BCTs for collecting and storing clinical oncology specimens for up to 3 days, ensuring reliable ctDNA and mutation analysis [2].

Polatoglou et al. compared automated and manual methods for isolating cfDNA from the plasma of healthy individuals. Their results contribute to understanding the variability of preanalytical methods and their impact on cfDNA measurements. These insights are essential for the development and implementation of routine clinical tests [3].

Randeu et al. emphasize the importance of method optimization and standardization. Their study demonstrated that variations in preanalytical workflows for mitochondrial DNA (mtDNA) analysis, such as blood collection tube type, sample storage temperature, storage duration, mtDNA extraction method, and matrix type, can influence the statistical differences observed between healthy individuals and cancer patients [4].

Apart from preanalytical workflows, Haselmann et al. discuss other significant factors that pose challenges to the development and implementation of cfDNA-based assays in routine clinical care. These factors include the lack of standardization in analytical workflows, insufficient reference materials and quality controls, the limited validation of clinical utility, conflicting research results, and challenges faced by clinicians [5].

There is evidence suggesting that cfDNA could serve as valuable molecular markers for monitoring and predicting various biological and physiological states in athletes. However, conventional sampling methods such as blood withdrawal and fingertip collection can be impractical, uncomfortable, or met with low compliance. Haller et al. present the earlobe as a feasible alternative for sample collection in many cases, but they emphasize the need for further research before drawing conclusive results [6].

## 3. Biological Considerations for cfDNA Analysis

In addition to preanalytical variables, several biological factors confound and limit cfDNA analyses. Clonal hematopoiesis of indeterminate potential (CHIP), which refers to the occurrence of somatic mutations in blood or bone marrow cells in the absence of hematologic neoplasms, has, for example, recently emerged as a potential cause of biological noise in cfDNA-based assays. Specifically, CHIP-associated mutations detected in cfDNA may result in the misdiagnosis of malignancy. However, Roma et al. observed a negligible incidence of CHIP-derived KRAS mutations in the cfDNA in a cohort of hundreds of cancer patients [7].

In his review paper, Gregor Hoermann highlights the significant pathological and clinical relevance of CHIP beyond being a confounding factor or a cause of false-positive results in cfDNA assays. He discusses research indicating the correlation between CHIP and increased risks of cardiovascular disease and hematological malignancies [8].

In our review paper, we delve into the extensive network of biological, physiological, lifestyle, and environmental factors that influence the quantitative and qualitative characteristics of cfDNA in the human body and in biospecimens. We demonstrate how these factors can complicate cfDNA analysis and pose significant challenges to developing specific cfDNA-based assays. However, we also explore how an improved understanding of the relationships between cfDNA characteristics and various biological factors and processes may not only enable a wide range of clinical applications but also provide unprecedented opportunities to study genomic changes in diverse contexts [9].

## 4. Physico-Chemical Properties

### 4.1. Epigenetic Features of cfDNA

Progress in cfDNA research has been driven forward rapidly by systematic characterizations of specific DNA mutations across basic and clinical research settings. However, the limitations of hotspot DNA mutation detection assays are becoming increasingly evident. In this Special Issue, three comprehensive appraisals of the literature outline the limitations of DNA hotspot mutational profiling and present other sequence and physico-chemical features of cfDNA as potential alternative or additive biomarkers.

Spencer Ding and Dennis Lo, who is renowned for his contributions to non-invasive prenatal testing (NIPT) and cfDNA fragmentomics, review several newly discovered features of cfDNA fragmentation, including fragment sizes, preferred ends, end motifs, single-stranded jagged ends, and nucleosomal footprints. They propose that these diverse cell- and disease-specific cfDNA fragmentation features can serve as surrogate markers for various disease indications. By highlighting the potential of both known and yet-to-be-discovered fragmentation features, they advocate for the expansion of the repertoire of diagnostic tools across a broad spectrum of diseases [10].

Oberhofer et al. also discuss cfDNA fragmentomics, but they also explore exciting and rapidly expanding research showing that various other epigenetic and physico-chemical features of cfDNA, such as various types of DNA methylation patterns, post-translational histone modifications, and nucleosome compaction patterns, are often cell- and disease-specific, which can be leveraged to trace the origins of cfDNA molecules. In their review, they focus particularly on the technical details of the methods and bioinformatics pipelines involved in the measurement of these various epigenetic features of cfDNA but also discuss the diagnostic implications and challenges involved in translating epigenetic measurements into clinically meaningful tools [11].

Variations in repetitive DNA are a hallmark of cancer and typically occur on a much wider scale across the genome vs. hotspot DNA mutations. A higher incidence across the genome corresponds with an increased incidence in cfDNA molecules, which in turn increases the probability of capturing mutant molecules and may significantly increase the analytical sensitivity and specificity of assays. Thus, the characterization of repetitive DNA sequences in cfDNA may be a powerful diagnostic approach. Bearing in mind that it is an underrepresented branch of research in the field, Gezer et al. summarize numerous research papers that have demonstrated the potential clinical value of profiling repetitive DNA elements [12].

### 4.2. Multi-Analyte Assessment

Beyond the characterization of various cfDNA features, cfDNA assays may be complemented by measurements of other analytes that provide similar or additive information about the disease in question. In characterizing exosomal and free-circulating miRNAs as potential biomarkers in colorectal cancer (CRC) patients, Dohmen et al. found that several exosomal miRNAs were enriched in CRC patients vs. healthy subjects. Although much clinical validation remains to be carried out, it is clear that exosomal miRNAs represent a unique class of liquid biopsy biomarkers that will receive an increasing amount of research attention in the coming years [13]. Based on parallel measurements of cfDNA and circulating tumor cells (CTCs) and extracellular vesicles (EVs) in cancer patients, Keup et al. highlight the clear-cut synergistic effects and combinatorial power of characterizing multiple different analytes present in one sample [14]. They emphasize the need for the further validation of multimodal tests and acknowledge that the logistical, technical, and bioinformatic requirements of integrative assays are not yet fully understood. Nevertheless, advancements in preanalytical workflows, assays, and technologies facilitating the simultaneous evaluation of multiple biomarker types within a single biospecimen are expected to greatly enhance the sensitivity and specificity of cfDNA-based assays. Ultimately, this will significantly improve the clinical utility of liquid biopsy assays as comprehensive diagnostic tools.

### 4.3. Structural Features of cfDNA

Rapidly growing interest in the characterization of the aforementioned physico-chemical features of cfDNA will catapult the field into a new era of intense research. This momentum has promise to not only accelerate the advent of personalized cancer care based on cfDNA assays but to also unlock the potential of cfDNA as a biomarker for various other diseases and clinical conditions. Despite some advancements, several fundamental properties of cfDNA molecules remain largely unknown, leaving numerous research questions to be explored.

For example, EV-associated DNA represents a unique population of cfDNA and likely contains additional information that can be mined for diagnostic purposes, or its biological relevance can be investigated. One of the hotly disputed topics related to EV-DNA that is still under investigation is whether the DNA exists primarily within the vesicle or whether it is localized on the exterior surface. With their experimental work on plasma exosomes, Tutanov et al. contribute to the body of evidence that DNA binds preferentially to the exterior surfaces of vesicles [15].

A prevalent bias in the research field suggests that a significant proportion of cfDNA molecules, particularly those derived from tumors, primarily comprise mono-nucleosomal cfDNA originating from apoptosis. However, accumulating evidence indicates that tumor-derived cfDNA exhibits diverse shapes and sizes and originates from multiple processes. In line with this, experiments by Ungerer et al. on cell culture models provided evidence that (i) both necrosis and apoptosis may govern the release of cfDNA, (ii) a significant portion of long cfDNA fragments generated via necrosis or other processes may be degraded into shorter fragments and rapidly feed the pool of mono-nucleosomes. This study also reports additional insights into the structures and compositions of cfDNA molecules [16].

Neutrophil extracellular traps (NETs) and neutrophil-derived EVs represent major sources of cell-free nucleic acids, and research suggests that these unique nucleic acids may have significant biological significance and clinical utility. However, NETs and neutrophil-derived EVs are currently significantly underappreciated in the field, and much is still not known about their compositions, biological properties, functions, and dynamics in the extracellular space. Heiko Pfister provides a timely and thorough review of various aspects related to NETs and neutrophil-derived EVs [17].

## 5. Clinical Applications

The potential clinical utility of cfDNA is now well understood, and the scope of potential clinical applications is expanding rapidly. Reviews and original research published in this Special Issue provide evidence for the clinical utility of various cfDNA biomarkers in various contexts.

Pesta et al. provide a detailed review of the clinical utility of cfDNA as a potential surrogate marker for various stages of non-small-cell lung cancer [18].

In recent years, next-generation DNA sequencing has become the go-to method for the high-sensitivity characterization of cfDNA. In their review paper, however, Gezer et al. outline evidence that DNA mutational profiling using droplet digital PCR (ddPCR) may be sufficiently sensitive as a liquid biopsy assay for the management of specific breast cancer patients [19].

In their study, Rivas-Delgado et al. profiled gene mutations and copy-number variations in the cfDNA of 20 patients with primary mediastinal large B-cell lymphoma (PMBL). They found a high concordance in the mutational profiles between cfDNA and tissue analysis, indicating that cfDNA may be an ideal biomarker and potentially an alternative method to tissue analysis for the management of PMBL cases [20].

In the work reported in their research article, Arellano et al. determined the hypermethylation status of the Septin 9 gene in the cfDNA of CRC patients before and several time-points after surgery. They found that the status of Septin 9 methylation in cfDNA can be used to detect residual disease after curative surgery and may potentially be used to predict recurrence [21].

In their study, Schmidt al. aimed to evaluate the usefulness of measuring methylated PTGER4 and SHOX2 plasma cfDNAs as biomarkers for monitoring therapy in lung cancer patients. They also investigated whether blood samples collected in stabilizing tubes could be processed at a later time. The baseline methylation levels of PTGER4 and SHOX2 did not differentiate between response groups initially. However, combining the methylation values of both genes enabled a clear differentiation between responders and non-responders during re-staging. Additionally, using stabilizing tubes for blood collection provided greater flexibility in the research process. These findings have implications for the development of more effective management strategies for lung cancer patients, particularly in therapy monitoring [22].

Trulson et al. evaluated cfDNA as a biomarker for estimating the severity and prognosis of trauma patients. They measured cfDNA levels in the plasma and serum of 164 patients upon admission to the emergency room, including those with severe trauma, moderate trauma, and single fractures. The results showed that cfDNA levels were significantly higher in patients with severe multiple trauma compared to those with moderate trauma or single fractures. Plasma and serum cfDNA levels strongly correlated with each other. The combination of cfDNA and hemoglobin improved the accuracy in identifying patients with severe multiple trauma. Within the multiple trauma group, higher cfDNA levels were observed in more severely injured patients and in those with traumatic brain injuries. Non-surviving patients had significantly higher cfDNA levels compared to survivors, and the combination of cfDNA, hemoglobin, and leukocytes enhanced the prognostic accuracy. This study indicates that cfDNA may become a valuable biomarker for estimating trauma severity and predicting the prognosis of trauma patients [23].

## 6. Cancer Screening via cfDNA

CfDNA molecules possess a unique set of features that make them ideal candidate biomarkers. Notably, they are cell-specific, continuously released from cells, possess short half-lives, and allow for non-invasive and serial sampling. Given that early detection and prompt treatment are paramount in the battle against cancer, the analysis of cfDNA profiles has the potential to revolutionize cancer patient management, propelling precision oncology toward comprehensive personalized medicine. Building upon this premise, Pons-Belda et al. [24,25] and Klein et al. [26] engage in a fruitful and open discourse, delving into critical aspects pertaining to the development and implementation of single- and multi-cancer screening tests. Their discussions encompass technical and analytical requirements, advantages, disadvantages, and challenges, as well as dispelling common misconceptions. As noted by Pons-Belda et al., various factors and challenges impede the sensitivity, specificity, and positive predictive value (PPV) of ctDNA screening tests. One prominent factor is the low abundance of ctDNA molecules in early-stage cancer. Despite these challenges, significant strides have been made to overcome these limitations, and many clinical trials using different approaches like methylation patterns or combinations of molecular and protein biomarkers are currently underway to validate the clinical utility of ctDNA screening tests. As highlighted by Klein et al., these novel pan-cancer screening approaches may help to overcome the shortcomings of the present cancer-screening paradigms, though large prospective screening trials are needed to validate the potential for reducing cancer-related mortality.

## 7. Concluding Remarks

This Special Issue encompasses diverse physico-chemical, pre-analytical, analytical, and clinical investigations concerning cell-free nucleic acids, particularly cfDNA. These articles offer valuable perspectives on the multifaceted nature of this new and highly informative analyte class, and we hope that they will stimulate further investigations aimed at comprehensively unraveling the biology and function of cell-free nucleic acids in human physiology and pathology. We anticipate that such research will be crucial for maximizing the full potential of cell-free nucleic acids in future medical diagnostics.

## References

[1] Gahan P.B., Schwarzenbach H., Anker P. (2022). The History and Future of Basic and Translational Cell-Free DNA Research at a Glance. Diagnostics.

[2] Diaz I.M., Nocon A., Held S.A., Kobilay M., Skowasch D., Bronkhorst A.J., Ungerer V., Fredebohm J., Diehl F., Holdenrieder S. (2023). Pre-Analytical Evaluation of Streck Cell-Free DNA Blood Collection Tubes for Liquid Profiling in Oncology. Diagnostics.

[3] Polatoglou E., Mayer Z., Ungerer V., Bronkhorst A.J., Holdenrieder S. (2022). Isolation and Quantification of Plasma Cell-Free DNA Using Different Manual and Automated Methods. Diagnostics.

[4] Randeu H., Bronkhorst A.J., Mayer Z., Oberhofer A., Polatoglou E., Heinemann V., Haas M., Boeck S., Holdenrieder S. (2022). Preanalytical Variables in the Analysis of Mitochondrial DNA in Whole Blood and Plasma from Pancreatic Cancer Patients. Diagnostics.

[5] Haselmann V., Hedtke M., Neumaier M. (2022). Liquid Profiling for Cancer Patient Stratification in Precision Medicine–Current Status and Challenges for Successful Implementation in Standard Care. Diagnostics.

[6] Haller N., Tomaskovic A., Stöggl T., Simon P., Neuberger E. (2022). Feasibility of Cell-Free DNA Measurement from the Earlobe during Physiological Exercise Testing. Diagnostics.

[7] Roma C., Sacco A., Forgione L., Esposito Abate R., Lambiase M., Dotolo S., Maiello M.R., Frezzetti D., Nasti G., Morabito A. (2022). Low Impact of Clonal Hematopoiesis on the Determination of RAS Mutations by Cell-Free DNA Testing in Routine Clinical Diagnostics. Diagnostics.

[8] Hoermann G. (2022). Clinical Significance of Clonal Hematopoiesis of Indeterminate Potential in Hematology and Cardiovascular Disease. Diagnostics.

[9] Bronkhorst A.J., Ungerer V., Oberhofer A., Gabriel S., Polatoglou E., Randeu H., Uhlig C., Pfister H., Mayer Z., Holdenrieder S. (2022). New Perspectives on the Importance of Cell-Free DNA Biology. Diagnostics.

[10] Ding S.C., Lo Y.D. (2022). Cell-Free DNA Fragmentomics in Liquid Biopsy. Diagnostics.

[11] Oberhofer A., Bronkhorst A.J., Uhlig C., Ungerer V., Holdenrieder S. (2022). Tracing the Origin of Cell-Free DNA Molecules through Tissue-Specific Epigenetic Signatures. Diagnostics.

[12] Gezer U., Bronkhorst A.J., Holdenrieder S. (2022). The Utility of Repetitive Cell-Free DNA in Cancer Liquid Biopsies. Diagnostics.

[13] Dohmen J., Semaan A., Kobilay M., Zaleski M., Branchi V., Schlierf A., Hettwer K., Uhlig S., Hartmann G., Kalff J.C. (2022). Diagnostic Potential of Exosomal microRNAs in Colorectal Cancer. Diagnostics.

[14] Keup C., Kimmig R., Kasimir-Bauer S. (2022). Combinatorial power of cfDNA, CTCs and EVs in oncology. Diagnostics.

[15] Tutanov O., Shtam T., Grigor’eva A., Tupikin A., Tsentalovich Y., Tamkovich S. (2022). Blood Plasma Exosomes Contain Circulating DNA in Their Crown. Diagnostics.

[16] Ungerer V., Bronkhorst A.J., Uhlig C., Holdenrieder S. (2022). Cell-Free DNA Fragmentation Patterns in a Cancer Cell Line. Diagnostics.

[17] Pfister H. (2022). Neutrophil Extracellular Traps and Neutrophil-Derived Extracellular Vesicles: Common Players in Neutrophil Effector Functions. Diagnostics.

[18] Pesta M., Shetti D., Kulda V., Knizkova T., Houfkova K., Sharif Bagheri M., Svaton M., Polivka J. (2022). Applications of Liquid Biopsies in Non-Small-Cell Lung Cancer. Diagnostics.

[19] Gezer U., Bronkhorst A.J., Holdenrieder S. (2022). The Clinical Utility of Droplet Digital PCR for Profiling Circulating Tumor DNA in Breast Cancer Patients. Diagnostics.

[20] Rivas-Delgado A., Nadeu F., Andrade-Campos M., López C., Enjuanes A., Mozas P., Frigola G., Colomo L., Sanchez-Gonzalez B., Villamor N. (2022). Cell-Free DNA for Genomic Analysis in Primary Mediastinal Large B-Cell Lymphoma. Diagnostics.

[21] Leon Arellano M., García-Arranz M., Guadalajara H., Olivera-Salazar R., Valdes-Sanchez T., García-Olmo D. (2022). Analysis of Septin 9 Gene Hypermethylation as Follow-Up Biomarker of Colorectal Cancer Patients after Curative Surgery. Diagnostics.

[22] Fleischhacker M., Arslan E., Reinicke D., Eisenmann S., Theil G., Kollmeier J., Schäper C., Grah C., Klawonn F., Holdenrieder S. (2023). Cell-Free Methylated PTGER4 and SHOX2 Plasma DNA as a Biomarker for Therapy Monitoring and Prognosis in Advanced Stage NSCLC Patients. Diagnostics.

[23] Trulson I., Stahl J., Margraf S., Scholz M., Hoecherl E., Wolf K., Durner J., Klawonn F., Holdenrieder S. (2023). Cell-Free DNA in Plasma and Serum Indicates Disease Severity and Prognosis in Blunt Trauma Patients. Diagnostics.

[24] Pons-Belda O.D., Fernandez-Uriarte A., Diamandis E.P. (2022). Multi Cancer Early Detection by Using Circulating Tumor DNA—The Galleri Test. Reply to Klein et al. The Promise of Multicancer Early Detection. Comment on “Pons-Belda et al. Can Circulating Tumor DNA Support a Successful Screening Test for Early Cancer Detection? The Grail Paradigm. *Diagnostics* 2021, *11*, 2171”. Diagnostics.

[25] Pons-Belda O., Fernandez-Uriarte A., Diamandis E. (2021). Can Circulating Tumor DNA Support a Successful Screening Test for Early Cancer Detection? The Grail Paradigm. Diagnostics.

[26] Klein E.A., Beer T.M., Seiden M. (2022). The Promise of Multicancer Early Detection. Comment on Pons-Belda et al. Can Circulating Tumor DNA Support a Successful Screening Test for Early Cancer Detection? The Grail Paradigm. *Diagnostics* 2021, *11*, 2171. Diagnostics.

